# Salivary Microbiota Composition in Patients with Oral Squamous Cell Carcinoma: A Systematic Review

**DOI:** 10.3390/cancers14215441

**Published:** 2022-11-04

**Authors:** Rodolfo Mauceri, Martina Coppini, Davide Vacca, Giorgio Bertolazzi, Vera Panzarella, Olga Di Fede, Claudio Tripodo, Giuseppina Campisi

**Affiliations:** 1 Department of Surgical, Oncological and Oral Sciences (Di.Chir.On.S.), University of Palermo, 90127 Palermo, Italy; 2 Tumor Immunology Unit, Department of Sciences for Health Promotion and Mother-Child Care “G. D’Alessandro”, University of Palermo, 90127 Palermo, Italy; 3 Department of Economics, Business, and Statistics, University of Palermo, 90128 Palermo, Italy

**Keywords:** microbiota, squamous cell carcinoma of head and neck, OSCC, dysbiosis, next-generation sequencing, NGS, periodontitis, *Porphyromonas*, *Fusobacterium*

## Abstract

**Simple Summary:**

This review aimed to analyse the current knowledge regarding the composition of salivary microbiota of patients with oral squamous cell carcinoma (OSCC). The protocol for this study was designed following the PRISMA guidelines. Observational studies, in human subjects with histological diagnosis of OSCC, concerning the analysis of salivary microbiota, were selected. Eleven papers were included. The salivary microbiomes of 1335 patients were analysed. Periodontal pathogens were the most frequent bacteria detected in patients with OSCC. We have found that although there are evident alterations in the composition of the salivary microbiota in OSCC patients, due to the great heterogeneity of the studies, it is still a challenge to identify a specific microbiota pattern. If the associations between alterations in the salivary microbiome and OSCC are confirmed, microbiome analysis could represent a useful tool for the screening and follow-up of patients affected by OSCC.

**Abstract:**

Background: Oral squamous cell carcinoma (OSCC) is one of the most prevalent cancers worldwide. Despite recent advances in diagnosis and treatment, in recent years, an increase in the incidence of OSCC has been registered, and the mortality rate is still high. This systematic review aims to identify a potential association between the composition of salivary microbiota and OSCC. Materials and Methods: The protocol for this study was designed following the PRISMA guidelines. Records were identified using different search engines (e.g., Medline/PubMed). Observational studies, in human subjects with histological diagnosis of OSCC, concerning the analysis of salivary microbiota, were selected. Results: Eleven papers were included. The salivary microbiomes of 1335 patients were analysed (n.687 OSCC and n.648 controls). Due to the great heterogeneity of the studies, it was not possible to profile a specific microbiota associated with OSCC. However, periodontal pathogens were the most common bacteria detected in patients with OSCC (i.e., *Fusobacterium*, *Prevotella*). Conclusions: Although there are evident alterations in the salivary microbiota composition in OSCC patients, it is still a challenge to identify a specific microbiota pattern in OSCC patients. If the associations between specific salivary microorganisms and OSCC are confirmed, microbiome analysis could be a useful tool for the screening and follow-up of patients affected by OSCC.

## 1. Introduction

In recent years, an increase in the incidence of oral cancer has been reported, and in 2020, approximately 377,713 new cases were diagnosed worldwide [1]. More than 90% of oral cancer is oral squamous cell carcinoma (OSCC) [2,3]. Despite recent advances in the diagnosis and treatment of OSCC, the overall 5-year survival rate of OSCC is still around 50–60% [4].

Despite the easy accessibility of the oral cavity, most cases of OSCC are diagnosed in advanced stages [2]. The etiopathogenesis of OSCC is multifactorial, and it has been related mainly to chemical factors (e.g., tobacco, alcohol) and also to other factors, such as infections (e.g., human papillomavirus) and genetic alterations [5,6].

The roles of microorganisms have been demonstrated in modulating and maintaining the biological functions of the human host. When the balanced relationship between microorganisms and the human host is disrupted (i.e., dysbiosis), either by external agents (e.g., smoking habits, alcohol consumption) or by infections of pathogenic microorganisms, the risk of developing a disease, including OSCC, may increase [7,8].

The term “*microbiota*” indicates all the microorganisms found in an environment and it includes bacteria, viruses, and fungi. The term “*microbiome*” defines the collective genome of microorganisms that reside in a particular environment and especially the human body (e.g., skin, gastrointestinal tract) [9].

The oral cavity is a dynamic and complex ecosystem, in which approximately 700 distinct prokaryotic taxa have been described [10,11]. Notably, virus aggregation has rarely been described in the human oral cavity compared to its bacterial counterpart [12].

Numerous studies demonstrated the relationship between human microbiota and carcinogenesis. The relationship between gut microbiota and tumorigenesis in the digestive region has been extensively investigated; for example, *Helicobacter pylori* has been shown to be associated with an increased risk for the development of gastric cancer [13]. *Salmonella typhi* and *Fusobacterium* have been associated with gallbladder and colon cancer, respectively [14,15,16].

The relationship between microbiota and OSCC onset may be related to chronic inflammatory processes, direct antiapoptotic effects, and the production of carcinogenic metabolites [17,18,19]. To date, the understanding of the role of oral microbiota and its implications in health and disease is limited and needs more evidence.

The oral cavity is subject to continuous changes in the composition of the ecological community that populate it. So, detecting changes in microbiota composition could represent a useful tool for the screening and follow-up of patients affected by OSCC [20].

Saliva offers an inexpensive, non-invasive, and easily accessible instrument to investigate possible associations between some patterns of salivary microbiota and OSCC [21,22].

Several saliva analyses have been proposed in oral medicine, such as oral rinse for HPV infection and salivary biomarkers for the potential signature of OSCC [23,24,25].

The aim of this systematic review is to identify a possible association between oral dysbiosis and OSCC, with particular regard to the studies that analysed only the salivary microbiota.

## 2. Materials and Methods

### 2.1. Protocol

A systematic literature search was conducted independently by two authors (MC and RM). The protocol for this study was designed following the Preferred Reporting Items for Systematic Reviews and Meta-Analyses (PRISMA) guidelines [26].

### 2.2. PICo and Research Question

The research question was designed based on PICo items in which:

P: patients affected by OSCC

I: alterations of salivary microbiota composition

Co: worldwide

The systematic review was based on the following research question: Do OSCC patients have alterations in the composition of their salivary microbiota?

### 2.3. Data Sources and Search strategy

A selection of studies concerning the analysis of salivary microbiota of patients affected by oral cancer was performed. Records were identified using different search engines (e.g., Medline/PubMed) and by scanning references lists of articles. For the search strategy, MeSH terms and free text words were combined through Boolean operators as follows: Microbiota AND ((oral cancer) OR (Squamous Cell Carcinoma of Head and Neck) OR (oral carcinoma)). Research was completed in January 2022.

### 2.4. Eligibility Criteria

The inclusion criteria for the studies were as follows:

Human studies;

- English language;
- At least 30 patients for study;
- Only diagnosis of OSCC histologically confirmed with a well-defined site classification;
- Only saliva analysis;
- Analysis of salivary microbiota in patients affected by OSCC, before therapy.

Exclusion criteria were: studies focused on tumours different from OSCC; narrative and systematic reviews and meta-analyses; case reports and studies with less than 30 patients; in vitro studies; in silico studies; animal studies; analysis of oral microbiome in patients affected by OSCC, during or after cancer therapy; studies with H&N or OP-SCC, no defined information for subgroup of OSCC; supragingival plaque analysis and tongue swab analysis; oral scraping.

### 2.5. Study Selection and Data Collection Process

**Figure 1:**
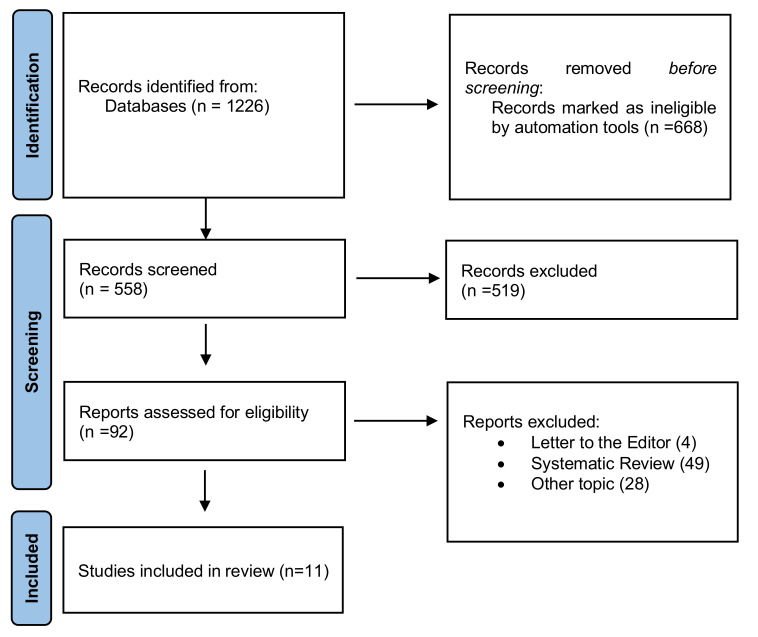
PRISMA 2020 flow chart.

The initial search strategy identified 1226 records, of which based on the inclusion criteria 668 studies were excluded. The screened process of 558 studies was performed based on the title and abstract, and 510 records were excluded. Subsequently, a full-text evaluation of 92 studies was carried out. Finally, 81 records were excluded, and 11 papers were included in the current review; a detailed flow chart of the selection process is provided in Figure 1.

### 2.6. Statistical Analysis

Selected studies were reviewed to detect outcomes of interest. For each study, the following data were extracted using a pre-designed data extraction Excel sheet. The following parameters were collected:

i. Study characteristics: name of the first author, year of publication, name of the country where the study was performed, study design.
ii. Case-control groups characteristics: case population, age of case population, control population, age of control population, risk factors.
iii. Methodology: sample collection, methods of sample collection, methods of DNA extraction, DNA amplification, sequencing platforms, reference database.
iv. Outcomes: raw reds detected, microbial abundance, genera detected, species detected, phyla detected, and OTUs detected.

Some results were not present in all the studies included in the review. Continuous variables were summarized with mean values and standard deviations, while categorical variables were expressed as counts and percentages.

## 3. Results

### 3.1. Study Characteristics

Eleven articles were included for this review. The main characteristics of the selected studies are described in Table 1 and Table 2. In detail, the characteristics of the included studies and the methodology employed for the analysis of the salivary microbiota are reported in Table 1; the exclusively statistically significant abundance of phyla, genera, and species in the study group are summarized in Table 2 (all results subdivided into phyla, genera, and species are described in Supplementary Materials).

All the articles included were observational studies published between 2017 and 2021. However, a wide heterogeneity was observed which made it impossible to perform an advanced statistical analysis. Eight studies were case–control [27,28,29,30,31,32,33,34], two were cohort studies [35,36], and one was a cross-sectional cohort study [37]. Of the eleven studies, five were from Taiwan [30,34,35,36,37], three were from China [28,29,32], one was from the USA [27], one was from Sudan [31], and one was from Japan [33].

Based on the available data, the average age of the patients ranged from 25 to 87 years [28,29,31,38], with a mean age of 56.8 ± 7.1 years [31,32,33,35].

Only four studies included both genders (179 male and 113 female) [28,29,31,33], four studies analysed exclusively the salivary microbiota of male patients (n. 534) [34,35,36,37], and in three studies the gender of the patients is not specified [27,28,30].

In total, the oral microbiomes of 1335 patients were analysed, of which 687 were affected by OSCC and 648 were controls. Among the control groups, 480 were healthy patients, 153 were affected by oral potentially malignant disorders, and 15 suffered from periodontitis.

Four studies compared salivary samples of patients affected by OSCC with healthy controls [28,31,33,34]. One study analysed salivary sample of patients affected by OSCC without a control group [35]. Three studies compared the oral microbiome of patients affected by OSCC, healthy controls, and oral potentially malignant disorders (OPMD) [27,30,36]. One study compared the oral microbiomes of patients affected by OSCC, healthy controls, and patients affected by gingivitis or periodontitis [29]. Two studies analysed the microbiome of cancer lesion and of anatomically matched normal sites of the same patients [32,37].

### 3.2. Sample Collection

The main information about the salivary collection and analysis technique is described in detail in Table 1. Generally, participants were instructed to refrain from eating, drinking, smoking, or oral hygiene prophylaxis for 30 min to 2 h before the saliva collection. In one study, the participants were asked not to take in any food and not brush or floss for at least 12 h before the sample collection [28]. Saliva samples were collected after mouth rinsing with 10 mL of sterile saline for 30 s. Salivary samples were collected by different techniques, such as sputum [30,31,33,34,35], oral rinse [27], and mucosa swab [32,37]. Besides saliva, some authors have also analysed subgingival plaque, healthy, and tumour tissue [28,29,38]. All studies analysed unstimulated saliva, except one study in which the participants were asked to chew gum for 5 min before salivary collection [33]. After collection, all samples were stored frozen at −80 °C [28,29,30,31,32,33,34,36,38,39], except in one study where the swab collections were stored at −20 °C [37].

### 3.3. DNA Extraction and Amplification

The different types of commercial DNA kits used were: QIAamp^®^ DNA Blood Mini Kit, QIAamp^®^ MinElute Virus Spin Kit, Modified QIAGEN^®^ DNA extraction method, FastDNA™ Kit, Gene Prep Star PI-80X device, E.Z.N.A.^®^ soil DNA Kit, and QIAampFast DNA Stool Mini Kit. DNA amplifications were carried out by targeting different hypervariable REGIONS of bacterial 16S rRNA genes. Few studies focused only on a single variable region such as V4 [30,35,37], while others focused on multiple regions, for instance V3–V4 [27,29,33,36], V3–V5 [34], and V4–V5 [28,32].

### 3.4. DNA Sequencing and Analysis of Data

After DNA amplification was completed, DNA sequencing was implemented. Eleven studies carried out sequencing by using the Illumina MiSeq System^®^. Just one study analysed different fungal genera using 454 GS FLX^®^ [31]. The processed sequencing reads were clustered into operational taxonomic units (OTU), and taxonomy classification was assigned according to the information retrieved from different databases (e.g., Greengenes database, SILVA database).

Finally, the functional composition of metagenomes was predicted from 16S rRNA data by the PICRUSt on five studies, and by PICRUSt2 software on one study [27,29,32,35,36,37].

### 3.5. Microbial Abundance

The main data regarding microbial abundance are described in detail in Table 2.

At the phylum level, only two studies defined the number of phyla detected, 11 and 12 phyla, respectively [27,32]. The most abundant phyla identified in the OSCC group were *Firmicutes*, *Bacteroidetes*, *Proteobacteria*, *Fusobacteria*, and *Actinobacteria* compared to healthy controls [30,35,37].

In the salivary samples of patients affected by OSCC, a significant abundance of *Spirochaetes*, *Fusobacteria,* and *Bacteroidetes* and a decrease in *Firmicutes* and *Actinobacteria* was observed by Zhao et al. [32].

Additionally, in the OSCC patients when compared to healthy controls, a decrease in *Actinobacteria* and *Fusobacteria* was reported by Su et al. and Yang et al., respectively [35,37].

With respect to genera, four studies defined the number of genera; the average number ranged from 36 to 130, with a mean number of 91.7 ± 36 genera detected [27,31,32,33].

In several studies, *Fusobacterium* was identified as the most abundant genera in OSCC patients, when compared to control groups [27,28,29,30,32,33,34,35,37,38]. Additionally, a high level of *Prevotella* and *Alloprevotella* was observed in various studies in patients affected by OSCC [27,33,35,37].

A decrease in *Streptococcus* in the OSCC group compared to healthy controls was reported in several studies [27,37].

Zhao et al. observed a significant abundance of 17 different taxa in the OSCC samples compared to the control group (see Supplementary Materials) [32], while the taxa *Megasphaera*, *Stomatobaculum*, *Granulicatella*, *Lautropia*, *Veillonella*, *Streptococcus*, *Scardovia*, *Rothia*, and *Actinomyces* were observed to be remarkably prevalent in the control group [32].

Additionally, Su et al. reported a significant increase in *Fusobacterium*, *Peptostreptococcus*, *Campylobacter*, *Prevotella*, and *Capnocytophaga* in the OSCC group [37].

Lee at al. reported the prevalence of *Prevotella*, *Veillonella*, *Streptococcus*, *Neisseria*, *Rothia*, *Fusobacterium*, *Haemophilus*, *Actynomices*, and *Porphyromonas* in all groups and the exclusive presence of *Megasphaera* in healthy controls, Escherichia in the PMD group, and *Atopobium* in the OSCC group [30].

Notably, the prevalence of *Capnocytophaga* was observed in relation to the advanced stage of OSCC by Yang et al. [35]. *Capnocytophaga* was observed to be significantly more abundant in the saliva of patients with a recurrence of OSCC also by Ganly et al. [27]. Moreover, Ganly et al. observed a high level of *Veillonella* and *Fusobacterium* in patients suffering from PMD lesions [27].

Takahashi et al. detected a significant abundance of *Streptococcus* and a low level of *Haemophilus* in female subjects compared to male subjects in the OSCC group [33].

A low level of *Rothia*, *Haemophilus*, and *Porhyromonas* was described in OSCC patients by Takahashi et al. and Yang et al. [33,35].

In healthy patients, Zhou et al. observed the exclusive presence of *Rothia* and high level of *Streptococcus*, *Neisseria*, *Prevotella*, *Porphyromonas*, and *Haemophilus* compared to OSCC groups [28].

Lastly, the fungal composition of oral microbiome was analysed only by Mohamed et al., who observed that *Candida* and *Saccharomyces* were more abundant in the saliva of OSCC patients, while *Cyberlindnera* was more abundant in healthy controls. Moreover, *Candida* was observed to be more abundant in female patients than in males [31].

In relation to species, only three studies reported specific data; the average number ranged from 172 to 389, with a mean number age of 253.7 ± 96.3 [27,32,34].

Zhao et al. reported 14 different species, defined as the oral mucosal core bacteriome of the OSCC patients (see Supplementary Materials) [32] and described an increase in *Neisseria flavescens* and *Fusobacterium periodonticum* in OSCC patients compared to healthy controls.

Su et al. observed a significant increase in *Campylobacter* spp. and a decrease in *Streptococcus pneumoniae* in OSCC patients compared to healthy controls [37].

An abundance of periodontal pathogens in the OSCC patients compared to healthy controls was observed in two studies: Li et al. reported an abundance of *Fusobacterium nucleatum* and *Porphyromonas gingivalis* [29], and Hsiao et al. of *Prevotella tannaerae*, *Fusobacterium nucleatum, and Prevotella intermedia* [34]. Hsiao et al. observed a decrease in *Streptococcus tigurinus* in the OSCC cohort compared to the healthy one [34].

Chen et al. reported a high level of Capnocytophaga sputigena, Catonella morbi, Prevotella oris, Peptostreptococcus stomatis, and Parvimonas micra in the OSCC patients, and of Veillonella parvula, Rothia dentocariosa, Porphyromonas gingivalis, and Tannerella forsythia in the OVH cohort [36], while in the control group, an abundance of Streptococcus pneumoniae was observed [37].

Mohamed et al. observed the presence of several species of *Candida*, *Malassezia*, and *Saccharomyces cerevisiae* in all salivary samples, while *C. orthopsilosis* and *C. sake* were identified exclusively in the saliva of OSCC patients [31].

Regarding the association between salivary microbial composition and other clinical factors, only three authors investigated it. Hsiao et al. detected several important associations: between higher levels of *P. intermedia* and alcohol drinking, and betel quid chewing, and between higher percentage of *F. nucleatum* and cigarette smoking. Moreover, it was observed that tooth brushing less than two times per day and a lack of regular dental visits were both associated with a significantly higher percentage of *P. tannerae*, while no use of dental floss was associated with a significantly higher percentage of *F. nucleatum* [34]. Additionally, a high prevalence of *Haemophilus* in patients who drink was observed by Takahashi et al. [33].

Chen et al. reported the association between HPV infection and *Haemophilus* and *Gemella* [36].

Mohamed et al. detected the presence of *Lodderomyces* only in the saliva of smokers, while *Phlebiopsis* and *Filobasidium* were detected only in the saliva of non-tobacco users. Furthermore, in the same study, it was reported that OSCC patients with locoregional lymph node involvement showed a higher abundance of *Candida* and *Aspergillus* and a lower abundance of *Malassezia* compared to the group with no lymph node involvement [31].

In the salivary samples of healthy patients, an abundance of Streptococcus, Neisseria, Prevotella, Porphyromonas, Haemophilus, and Rothia was reported by Zhou et al. [28]

Lee et al. observed the presence of the genus Megasphaera exclusively in the healthy group [30].

Regarding the fungi composition, Mohamed et al. reported the abundance of M. arunolokei in the salivary microbiota of healthy patients [31].

With regard to the main phyla, genera, and species reported as statistically significant associated with OSCC presence, it was not possible to profile a specific microbiota associated with OSCC. However, based on the present findings, periodontal pathogens (e.g., *Fusobacterium*, *Prevotella*) were the most common bacteria detected in patients with OSCC.

## 4. Discussion

OSCC is one of the most prevalent cancers worldwide [40]. Despite recent advances in diagnosis, surgical techniques, and adjuvant therapy, in recent years, an increase in the incidence of OSCC has been registered, and the mortality rate is still high [41]. OSCC is of multifactorial origin; several factors act individually or in combinations in the pathogenesis. Most of the risk factors that have been identified are lifestyle related; however, about 15% of OSCC is not associated with any known risk factors [42].

Regarding the potential role of oral microbiota in the carcinogenesis process, very few studies have investigated the salivary microbiota composition in patients affected by OSCC compared to healthy control.

Unlike the study of the microbiome of different regions of the body, the oral one possesses some criticalities. Indeed, oral microbiota is a complex dynamic ecological community conditioned by continuous changes in the availability of oxygen, nutrients, and the pH of saliva, since it contains very distinct niches adhering to various surfaces [18]. According to the Human Oral Microbiome Database (HOMD), only approximately 57% of the oral bacterial species were identified, 13% were cultivated but they remain nameless, and 30% are neither isolable nor replicable in cultures microbiological [43].

This review aimed to analyse the current knowledge regarding the composition of salivary microbiota of patients with OSCC and the possible association between salivary changes and OSCC.

Eleven studies were included from different parts of the world, most of these from Asia. Although all studies analysed oral microbiota composition of patients affected by OSCC, different techniques of saliva collection, DNA extraction, and data analysis were employed.

The most common technique for saliva collection is sputum, followed by mucosa swab, and oral rinse.

There are more selective methods to analyse the microorganisms that colonize specific oral surfaces separately (e.g., tongue, buccal mucosa) [18]. However, the procedures required are often complicated, since it is difficult to collect saliva from a determined surface with precision and selectively [44].

To evaluate microbiota composition from the whole saliva, different methods have been developed. Currently, the study of the oral microbiota is based on the sequencing of the genome (next-generation sequencing, NGS). This technology is mainly employed to determine the order of nucleotides in entire genomes or targeted regions of DNA or RNA. This methodology to characterize the microbiome structure is probably the most utilized because it is relatively quick and easy [45].

The two most used approaches of NGS technique are the *shotgun* metagenome sequencing, which analyses the entire metagenome, and sequencing of the 16S ribosomal RNA amplicon, generally 16S rRNA for bacteria and 18S rRNA/ITS for fungi. Both techniques read the DNA sequences of the microbes present in a sample comparing them with a sequence database to establish relative quantities of different organisms present in that sample [18,46]. In almost all the included studies, the sequencing of 16S ribosomal RNA amplicon was the method applied. All these studies used the Illumina MiSeq System^®^, except one study that analysed fungal genera composition, using the 454 GS FLX^®^ [31].

Despite the great heterogeneity of the included articles, the most common genera detected in the OSCC patients were *Fusobacterium* and *Prevotella*, and in some studies, *Streptococcus.* Interestingly, these three bacteria are periodontal pathogens: *Fusobacterium* and *Prevotella* are Gram-negative anaerobic pathogens, and *Streptococcus* is a Gram-positive facultative anaerobic pathogen.

We believe it is possible that these periodontopathic bacteria are directly and indirectly involved in oral carcinogenesis as they participate in three mechanisms: the development of chronic pro-inflammatory processes, direct anti-apoptotic action, and the production of carcinogenic metabolites [17,18,47].

The development of chronic pro-inflammatory processes, due to the presence of periodontal pathogenic bacteria (e.g., *Fusobacterium* and *Prevotella*), stimulates the production of several cytokines, including interleukin-1ß (IL-1ß) and IL-6, tumour necrosis factor-α (TNF-α), and matrix metalloproteinases (MMPs) [19,48]. IL-1ß promotes angiogenesis and cancer progression, stimulating bone resorption, production of vascular endothelial growth factor, and other pro-angiogenic mediators [49,50]. IL-6 alters the cell cycle suppressing apoptosis, stimulates the adhesion of tumour cells to endothelial cells, and promotes the process of tissue invasion and metastasis, increasing the expression of MMPs [51,52]. TNF-α generates DNA damage and stimulates carcinogenesis-promoting prostaglandin and pro-inflammatory cytokines production [51,53].

A direct anti-apoptotic action is stimulated by *P. gingivalis* and *F. nucleatum* infection. *P. gingivalis* stimulates the secretion of antiapoptotic factors (e.g., Bcl-2) and reduces the expression of the tumour suppressor gene *p53*, promoting the migration of cancer cells [53,54]. Additionally, *F. nucleatum* participates in tissue invasion and metastasis development, activating *p38* with the consequent secretion of MMP-9 and MMP-13 [19,55].

Lastly, the production of bacteria carcinogenic metabolites can also promote carcinogenesis through the release of free radicals in chronic inflammatory processes. Several *Streptococcus* species have been observed to be associated with the production of H_2_O_2_, NO_2_, and acids (e.g., lactic acid), which lower the tissue pH, contributing to the development of hypoxia in the tumour microenvironment and increasing the risk of metastasis [56,57]. Furthermore, thanks to the enzyme alcohol dehydrogenase, these bacteria metabolite alcohol into acetaldehyde, a carcinogenic substance [58]. Finally, *F. nucleatum*, *P. gingivalis*, and *P. intermedia* have the ability to produce volatile sulphur compounds (e.g., sulphuric acid), which promote the development and accumulation of genetic mutations [59].

Regarding the fungi potential involvement in oral carcinogenesis, only one included study investigated the fungi microbiota composition in patients with OSCC [31]. Fungi represent about 1% of the microbiota, and the genus *Candida* is the most easily isolated and, therefore, the most studied. *Candida* genus may be involved in OSCC onset through the production of potentially carcinogenic substances, such as endogenous nitrosamines starting from the nitrites present in the saliva [56]. Additionally, it has been observed that *C. albicans* promotes the adhesion of bacteria on the oral mucosa (e.g., *S. Mutans*), making bacteria more resistant to antibiotic treatments and consequently stimulating the creation of microbial infections, promoting chronic inflammation [60].

To be best of our knowledge, this is the first study that has attempted to compare the results from different studies to identify a potential association between a specific salivary microbiota and OSCC.

This review possesses some limitations derived from the great heterogeneity of the few studies present in the literature to date. Of these, some studies compared salivary samples of patients affected by OSCC with healthy controls, others analysed cancer lesions and anatomically matched normal sites of the same patients, and only one study compared the salivary samples of patients with OSCC with patients affected by periodontitis and healthy controls.

The wide variation of the results observed could be explained by two variables: the absence of a standardized protocol of oral microbiome analysis and the extreme heterogeneity of the oral microbiome composition associated with healthy and OSCC patients. Therefore, further investigations are required to identify and validate standardized protocols of microbiota analysis and to investigate its implication in oral carcinogenesis.

Even if saliva represents a very attractive sample because it is easily sampled, non-invasive, and reflects systemic conditions, to date the gold standard for OSCC diagnosis is still the incisional biopsy for histological confirmation [22,61]. However, if the associations between specific salivary microorganisms and OSCC are confirmed, microbiome analysis could be a useful screening test to identify “*high-risk*” patients. Indeed, we believe that in the near future, salivary microbiota analysis may be the litmus test for clinicians to intercept rapidly and easily the possible changes in saliva composition. Additionally, in the case of dysbiosis, saliva analysis could promote personalized approaches to restore the “good” oral microbiome and prevent oral diseases, as well as carcinogenesis, if its connection is proven. Consequently, these patients could undergo regular oral examinations and increase the frequency of check-ups to improve early diagnosis and focused therapy.

## 5. Conclusions

There is an increasing interest in the analysis of the saliva and oral microbiome composition to identify a potential association between oral dysbiosis and OSCC. However, although there are evident alterations in the composition of the salivary microbiota in OSCC patients, it is still a challenge to identify a specific microbiota pattern in OSCC patients. We encourage future standardized studies to increase the knowledge about salivary microbiota and its potential applications.

## Figures and Tables

**Table 1:** Characteristics of the included studies and the methodology employed for the analysis of the salivary microbiota.

N.	Author, Year	Country	Study Design	N. of Case	N. of Control	Sample	Methods of DNA Extraction	DNA Amplification	Sequencing	Reference Database
1	Lee WH, 2017 [25]	Taiwan	Case-control	125	251	Sputum	QIAamp^®^ DNA Blood Mini Kit	V4	Illumina MiSeq System	SILVA
2	Zhao H, 2017 [26]	China	Case-control	40	40 *	Oral swabs	QIAamp^®^ DNA Blood Mini Kit	V4–V5	Illumina MiSeq System	RDP
3	Hsiao JR, 2018 [24]	Taiwan	Case-control	138	151	Sputum	QIAamp^®^ MinElute Virus Spin Kit	V3–V5	Illumina MiSeq System	RDP
4	Yang SF, 2018 [27]	Taiwan	Cohort study	39	No control group	Sputum	QIAamp^®^ DNA Blood Mini Kit	V4	Illumina MiSeq System	Greengenes
5	Mohamed N, 2019 [28]	Sudan	Case-control	59	13	Sputum	FastDNA™ Kit	fungal ITS2 region	Illumina MiSeq System	UNITE
6	Takahashi Y, 2019 [29]	Japan	Case-control	60	80	Sputum	Gene Prep Star PI-80X device	V3–V4	Illumina MiSeq System	SILVA 128
7	Li Y, 2020 [30]	China	Case-control	10	30	Saliva, subgingival plaque, tumour and healthy surface	QIAampFast DNA Stool Mini Kit	V3–V4	Illumina MiSeq System	SILVA
8	Chen JW, 2021 [31]	Taiwan	Cohort study	27	48	Sputum	QIAamp^®^ DNA Blood Mini Kit	V3–V4	Illumina MiSeq System	HOMD
9	Ganly I, 2021 [32]	USA	Case-control	18	20	Ora rinse	Modified QIAGEN^®^ DNA extraction method	V3–V4	454 FLX platform	Greengenes
10	Su SC, 2021 [33]	Taiwan	Cross-sectional	116	116 *	Oral swabs	QIAamp^®^ DNA Blood Mini Kit	V4	Illumina MiSeq System	SILVA
11	Zhou X, 2021 [34]	China	Case-control	47	48	Saliva, subgingival plaque, tumour and healthy surface	E.Z.N.A.^®^ soil DNA Kit	V4–V5	Illumina MiSeq System	SILVA

* Studies that analysed contralateral normal regions of the same patients as control group.

**Table 2:** Description of the statistically significant results about phyla, genera, and species of the studies included.

Author, Year	N. of Phyla Detected	Phyla		N. of Genera Detected	Genera		N. of Species Detected	Species	
		↑ in OSCC Group	↓ in OSCC Group		↑ in OSCC Group	↓ in OSCC Group		↑ in OSCC Group	↓ in OSCC Group
Zhao H, 2017 [26]	11	Spirochaetes, Fusobacteria, Bacteroidetes	Firmicutes, Actinobacteria	130	Mycoplasma, Treponema, Campylobacter, Eikenella, Centipeda, Lachnospiraceae, Alloprevotella, Fusobacterium, Selenomonas, Dialister, Peptostreptococcus, Filifactor, Peptococcus, Catonella, Parvimonas, Capnocytophaga, and Peptostreptococcaceae	Megasphaera, Stomatobaculum, Granulicatella, Lautropia, Veillonella, Streptococcus, Scardovia, Rothia, and Actinomyces	389	n.d.	n.d.
Hsiao JR, 2018 [24]	n.d.	n.d.	n.d.	n.d.	n.d.	n.d.	120	*P. tannaerae* and *F. nucleatum*	n.d.
Yang SF, 2018 [27]	n.d.	n.d.	n.d.	n.d.	Capnocytophaga *	n.d.	n.d.	n.d.	n.d.
Takahashi Y, 2019 [29]	n.d.	n.d.	n.d.	85	Peptrostreptococcus, Fusobacterium, Alloprevotella, Capnocytophaga	Rothia, Haemophilus	n.d.	n.d.	n.d.
Ganly I, 2021 [32]	12	n.d.	n.d.	116	Fusobacterium, Prevotella, Alloprevotella	Streptococcus	172	n.d.	n.d.
Su SC, 2021 [33]	n.d.	n.d.	n.d.	n.d.	Fusobacterium, Peptostreptococcus, Campylobacter, Prevotella, Capnocytophaga	Streptococcus	n.d.	*Campylobacter* spp.	*Streptococcus pneumoniae*

* Statistically significant differences only in OSCC diagnosed at the late stage. ↑ phyla, genera, and species increased in the OSCC group. ↓ phyla, genera, and species reduced in the OSCC group

## Data Availability

Not applicable.

## Supplementary Materials

The following supporting information can be downloaded at: https://www.mdpi.com/article/10.3390/cancers14215441/s1, Table S1: Description of all the results about phyla, genera and species of the studies included.
