# Supplementary material for: Salivary Microbiota Composition in Patients with Oral Squamous Cell Carcinoma: A Systematic Review
Source: Cancers (Basel). 2022 Nov 4;14(21):5441. doi: 10.3390/cancers14215441 (PMC9656014; doi:10.3390/cancers14215441)
Supplement: Supplementary file 1 [file cancers-14-05441-s001.zip › cancers-2005698-supplementary.pdf]

cancers

Systematic Review

Salivary Microbiota Composition in Patients with Oral Squamous Cell Carcinoma: A Systematic Review

Rodolfo Mauceri [1,*], Martina Coppini [1], Davide Vacca [2], Giorgio Bertolazzi [2,3], Vera Panzarella [1], Olga Di Fede [1], Claudio Tripodo [2] and Giuseppina Campisi [1]

1 Department of Surgical, Oncological and Oral Sciences (Di.Chir.On.S.), University of Palermo, 90127 Palermo, Italy
2 Tumor Immunology Unit, Department of Sciences for Health Promotion and Mother-Child Care "G. D'Alessandro", University of Palermo, 90127 Palermo, Italy
3 Department of Economics, Business, and Statistics, University of Palermo, 90128 Palermo, Italy
* Correspondence: rodolfo.mauceri@unipa.it

Citation: Mauceri, R.; Coppini, M.; Vacca, D.; Bertolazzi, G.; Panzarella, V.; Di Fede, O.; Tripodo, C.; Campisi, G. Salivary Microbiota Composition in Patients with Oral Squamous Cell Carcinoma: A Systematic Review. *Cancers* **2022**, *14*, 5441. https://doi.org/10.3390/cancers14215441

Academic Editor: Miguel Angel González Moles

Received: 17 October 2022
Accepted: 2 November 2022
Published: 8 November 2022

Publisher's Note: MDPI stays neutral with regard to jurisdictional claims in published maps and institutional affiliations.

Copyright: © 2022 by the authors. Licensee MDPI, Basel, Switzerland. This article is an open access article distributed under the terms and conditions of the Creative Commons Attribution (CC BY) license (https://creativecommons.org/licenses/by/4.0/).

Table S1. Description of all the results about phyla, genera and species of the studies included.

N.	Author, Year	N. of phyla detected	Phyla		N. of genera detected	Genera		N. of species detected	Species
1	Lee WH, 2017 [25]	n.d.	Firmicutes, Bacteroidetes, Proteobacteria, Actinobacteria and Fusobacteria in all individuals, with minor variations among groups		n.d.	↑ in OSCC group: Atopobium*<br><br>↑ in PMD group: Escherichia*<br><br>↑ in healthy controls: Megasphaera*	n.d.	n.d.	n.d.
2	Zhao H, 2017 [26]	11	↑ in OSCC group: Spirochaetes, Fusobacteria, Bacteroidetes	↓ in OSCC group: Firmicutes, Actinobacteria	130	↑ in OSCC group: Mycoplasma, Treponema, Campylobacter, Eikenella, Centipeda, Lachnospiraceae_G_7, Alloprevotella, Fusobacterium, Selenomonas, Dialister, Peptostreptococcus, Filifactor, Peptococcus, Catonella, Parvimonas, Capnocytophaga, and Peptostreptococcaceae_ XI_G_7.	↓ in OSCC group: Megasphaera, Stomatobaculum, Granulicatella, Lautropia, Veillonella, Streptococcus, Scardovia, Rothia, and Actinomyces	389	↑ in OSCC group: P. melaninogenica , A. odontolyticus, P. scopos, C. gingivalis, G. sanguinis, G. adiacens, S. oralis, S. salivarius, L. umeaense, V. atypica, F. periodonticum, N. elongata, N. flavescens, H. parainfluenzae

3	Hsiao JR, 2018 [24]	n.d.	n.d.		n.d.	n.d.		200	↑ in OSCC group: P. tannaerae, F. nucleatum, P. intermedia	↓ in OSCC group: S. tigurinus
4	Yang SF, 2018 [32]	n.d.	↑ in OSCC group: Firmicutes, Bacteroidetes, Proteobacteria, Actinobacteria	↓ in OSCC group: Fusobacteria	n.d.	↑ in OSCC group: Streptococcus, Prevotella, Neisseria, Veillonella, and Capnocytophaga	↓ in OSCC group: Rothia, Porphyromonas.	n.d.	n.d.	
5	Mohamed N, 2019 [30]	n.d.	n.d.		36	↑ in OSCC group: Candida, Saccharomyces	↓ in OSCC group: Cyberlindnera	n.d.	↑ in OSCC group: S. cerevisiae, C. orthopsilosis* and C. sake*	↓ in OSCC group: M. arunolokei.
6	Takahashi Y, 2019 [31]	n.d.	n.d.		85	↑ in OSCC group: Peptrostreptococcus, Fusobacterium, Alloprevotella, Capnocytophaga	↓ in OSCC group: Rothia and Haemophilus	n.d.	n.d.	
7	Li Y, 2020 [28]	n.d.	n.d.		n.d.	↑ in OSCC group: Fusobacterium, Streptococcus, and Atopobium	↓ in OSCC group: Haemophilus and Rothia	n.d.	↑ in OSCC group: F. nucleatum, P. gingivalis	n.d.
8	Chen JW, 2021 [33]	n.d.	n.d.		n.d.	n.d.		n.d.	↑ in OSCC group: C. sputigena *, C.	n.d.

								morbi*, D. invisus*<br>P. oris, P. stomatis,<br>P. micra<br><br>↑ in OVH group:<br>V. parvula, R. dentocariosa,<br>A. geminatus*,<br>P.gingivalis*,<br>P. oulorum*, S. (TM7) [G5] bacterium HMT-356*, T. forsythia*, D. invisus*	
9	Ganly I, 2021 [29]	12	n.d.	116	↑ in OSCC group:<br>Fusobacterium,<br>Prevotella,<br>Alloprevotella.<br>Capnocytophaga.<br><br>↑ in PMD group:<br>Veillonella, Fusobacterium	↓ in OSCC group: Streptococcus	172	n.d.	

10	Su SC, 2021 [34]	n.d.	↑ in OSCC group: Firmicutes, Proteobacteria, Bacteroidetes, Fusobacteria	↓ in OSCC group: Actinobacteria	n.d.	↑ in OSCC group: Fusobacterium, Peptostreptococcus, Campylobacter, Prevotella, Capnocytophaga	↓ in OSCC group: Streptococcus.	n.d.	↑ in OSCC group: Campylobacter spp.	↓ in OSCC group: Streptococcus pneumoniae
11	Zhou X, 2021 [27]	n.d.	n.d.		n.d.	↑ in OSCC group: Actinobacteria, Fusobacterium, and Veillonella.	↑ in control group: Streptococcus, Neisseria, Prevotella, Porphyromonas, Haemophilus, Rothia*	n.d.	n.d.	

* Studies that analysed contralateral normal regions of the same patients as control group
